# Novel role of Snail 1 in promoting tumor neoangiogenesis

**DOI:** 10.1042/BSR20182161

**Published:** 2019-05-10

**Authors:** Yi-Kun Zhang, Hua Wang, Yu-Wei Guo, Yang Yue

**Affiliations:** 1 Department of Hematology, PLA Strategic Support Force Caracteristic Medical Center, Beijing 100101, China; 2 Department of Experimental Hematology, Beijing Institute of Radiation Medicine, Beijing 100850, China

**Keywords:** epithelial to mesenchymal transition, Neoangiogenesis, Snai1, tumor derived endothelial cells

## Abstract

Snail1 plays an important role in epithelial to mesenchymal transition (EMT) during tumor metastasis; however, whether Snai1 potentiates the process of neoangiogenesis is completely unknown. In the present study, tube formation assay was used to evaluate neoangiogenesis *in vitro*. The expression of Snai1 and other pro-neoangiogenic factors was measured by quantitative real time PCR. Tumor derived endothelial cells (TDECs) were stimulated with fibroblast growth factor 1 (FGF1) or VEGF and formed more tubes compared with untreated, whereas cells treated with Sulforaphane had less tube formation. Silencing *SNAI1* significantly attenuated tube formation accompanied by decreased CD31, CD34, and VWF expression in TDECs compared with control. In contrast, overexpression of Snai1 led to more CD31, CD34, and VWF expression and tube formation. To determine if the observed effects of *SNAI1* on tube formation was a global phenomenon, the same assay was conducted in normal mesenchymal stem cells (MSCs). *SNAI1* silencing did not have any effect on tube formation in MSCs. The expression of *TIMP2, ENG*, and *HIF1A* was up-regulated 3-fold or higher after silencing *SNAI1*, and *ID1, VEGFA, PLG, LECT1, HPSE* were shown down-regulated. Taken together, our study elucidates an important role of EMT inducer Snai1 in regulating tumor neoangiogenesis, suggesting a potential therapeutic target for overcoming tumor EMT.

## Introduction

Epithelial to mesenchymal transition (EMT) is a fundamental process in which epithelial cells undergo morphological changes to highly motile mesenchymal cells. EMT is an important pre-requisite for cancer metastasis, which is the cause for 90% of cancer-related mortality [1,2]. One of the major effector of EMTs is the zinc-finger factor Snai1 [3,4]. The gene encoding Snail 1, *SNAI1*, has been shown through extensive research work to be indispensable for cancer metastasis, largely due its direct role in inhibiting the epithelial cell marker, E-cadherin [5–8]. Tumor cells reside in an area of hypoxia and require neoangiogenesis to promote new blood vessel growth to sustain tumor growth and viability [9]. Numerous cytokines and signaling molecules including VEGF, fibroblast growth factor (FGF), hypoxia-inducible factor 1 α (HIF1α) have been implicated in neoangiogenesis. However, little is known about the contribution of the EMT program to neoangiogenesis. Given that the entire metastatic cascade is a continuing process, it can be envisaged that one process will feed into the following one. Hence, the objective of the current study was to evaluate whether EMT inducers impact the process of neoangiogenesis. As one of the hallmarks in EMT and metastatic progression [3–7,9], Snai1 was investigated to determine its contribution to the process of neoangiogenesis.

## Materials and methods

### Tumor derived endothelial cells

Human lung tumor derived endothelial cells were obtained from five independent donors from Cell Biologics (Chicago, IL, U.S.A.) and maintained in the EBM-2 endothelial growth basal medium (Lonza), supplemented with 5% FBS (Life Technologies, Shanghai, China) and 1% penicillin–streptomycin solution in a 5% CO_2_ atmosphere at 37°C.

The research has been carried out in accordance with the World Medical Association Declaration of Helsinki, and all subjects provided written informed consent.

### Mesenchymal stem cell isolation and culture

Mesenchymal stem cells (MSCs) were isolated from whole blood from five healthy donors (all enrolled in the study after signing on informed consent and post approval from our Institutional Review Board) with RosetteSep Human Mesenchymal Stem Cell Enrichment Cocktail (Stem Cell Technologies, Vancouver, BC, Canada). MSCs were cultured under the same conditions as TDECs.

### Stimulation and inhibition of TDECs

Where indicated, TDECs were treated with 20 µm Sulforaphane (Sigma–Aldrich, Shanghai, China) for 24 h, FGF (5 ng/ml) (PeproTech, Rocky Hill, NJ, U.S.A.) for 6 h, rVEGF (10 ng/ml) (PeproTech) for 12 h.

### Tube formation assay *in vitro*

Growth factor-reduced Matrigel in a 24-well format was used for the formation of tubular structures *in vitro*. Indicated cells (4 ×10^4^ cells/well) were seeded onto Matrigel-coated wells in EBM-2 medium with or without indicated additives. Tube formation was evaluated after 6 h culture with a Nikon inverted microscope. Images (×10 fields) were taken, and endothelial tubes were quantitated by counting length and branches with NIS Elements software [10].

### Western blotting

Whole-cell lysates were resolved on a denaturing 10% SDS-PAGE gel and subsequently transferred to polyvinylidene fluoride membranes via semidry transfer. After blocking the membrane at room temperature with 5% skim milk for 1 h, the membrane was incubated overnight at 4°C with anti-CD31 (1:1000, abcam), anti-CD34 (1:1000, abcam), anti-VWF (1:1000, abcam) antibodies. After incubation with peroxidase-conjugated secondary antibodies at a dilution of 1:2000 for 1 h and washed three times with PBS, the signals were visualized using enhanced chemiluminescence.

### Quantitative real time polymerase chain reaction

TRIzol reagent was used to isolate total RNA from cultured TDECs transfected with siRNA targetting *SNAI1* or Luciferase. The RevertAid™ First Strand cDNA synthesis Kit (Life Technologies, Shanghai, China) was utilized to synthesize the first strand cDNA. cDNA obtained from the patients was then used to template the Human Angiogenesis RT^2^ Profiler PCR Array (Qiagen, Beijing, China). The RT Profiler PCR Array profiles the expression of 84 key genes that either change their expression during angiogenesis or regulate those gene expression changes. Data were normalized to *GAPDH* expression and analyzed by the −ΔΔ*C*_T_ method.

### Overexpression and silencing of Snai1

According to the manufacturer’s instruction, TDECs or MSCs were transiently transfected with 50 nM of *SNAI1* siRNA (Silencer Select, Life Technologies) or pCMV-Snai1 (Silencer Select, Life Technologies) for 48 h using Lipofectamine 2000 Transfection Reagent (Life Technologies, Shanghai, China). The cells were also treated with a scrambled siRNA targetting Firefly luciferase or pCMV (Silencer Select, Life Technologies) as a negative control. The efficiency of Snai1 silencing or overexpression was determined by western blot.

### Statistical analysis

All experiments were repeated at least for three times. All data were present as means ± S.D. Statistical analysis was made by ANOVA test. Differences were considered significant at *P*<0.05.

## Results

**Figure 1:**
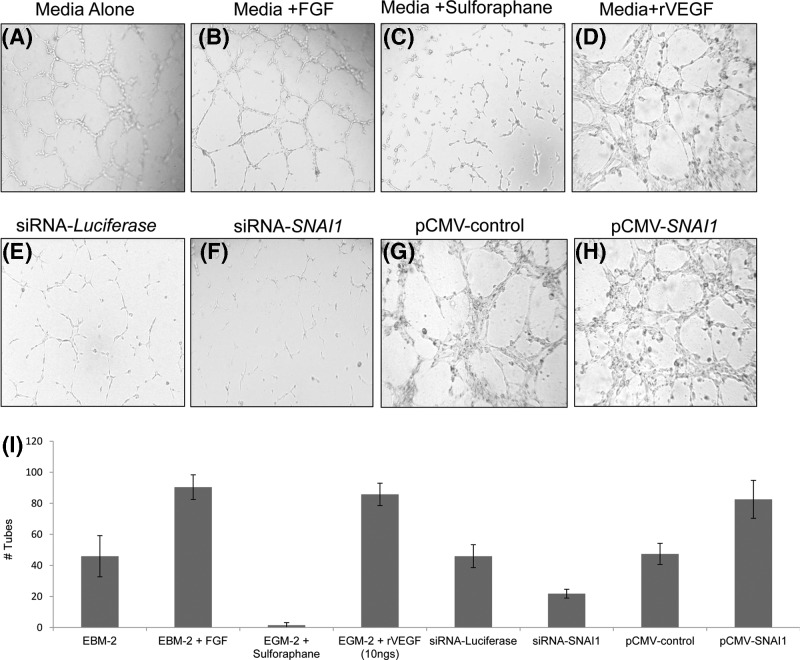
*SNAI1* expression is required for tube formation *in vitro* (**A–D**) TDECs were under different treatments and tested for neoangiogenesis by tube formation assay. (**E–H**) Tube formation was performed with different treatments in TDECs. Cells were transiently transfected with SNAI1 siRNA (siSNAI1) at a concentration of 50 nM and scrambled siRNA (luciferase) was used as a negative control. Otherwise, TDECs were transiently transfected with pCMV-SNAI1 at a concentration of 50 nM or control (pCMV). (**I**) Quantitation of tube formation in indicated conditions in all five donors-derived TDECs were shown. Data are present with means ± S.D. *P<0.05.

**Figure 2:**
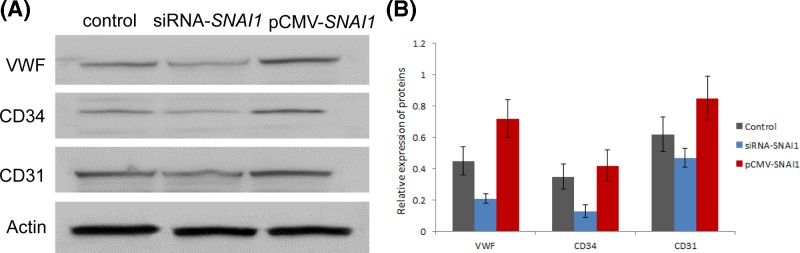
Snai1 increases expression of angiogenic markers in TDECs Cells were transiently transfected with SNAI1 siRNA (siSNAI1) at a concentration of 50 nM and scrambled siRNA (luciferase) was used as a negative control. Otherwise, TDECs were transiently transfected with pCMV-SNAI1 at a concentration of 50 nM or control (pCMV). Angiogenic activation markers CD31, CD34, and VWF were measured by western blot (**A,B**). Data are present with means ± S.D.

To evaluate neoangiogenesis *in vitro*, the tube formation assay was performed. TDECs were treated with FGF, VEGF, or Sulforaphane, and we found cells stimulated with FGF and VEGF formed more tubes than untreated, whereas Sulforaphane attenuated tube formation (Figure 1A–D). To estimate the role of Snai1 in neoangiogenesis, Snai1 gene was knocked down with small interfering RNA (siRNA). The Snai1 protein was down-regulated and tube formation (Figure 1E,F) as well as angiogenic activation markers CD31, CD34, and VWF (Figure 2) were also reduced after silencing *SNAI1* in TDECs after the treatment of FGF1 and VEGF). In contrast, overexpression of Snai1 led to more CD31, CD34, and VWF expression and tube formation (Figures 1G,H & 2). The quantitation results of TDEC-induced tube formationfrom all five donors were summarized in Figure 1I.

**Figure 3:**
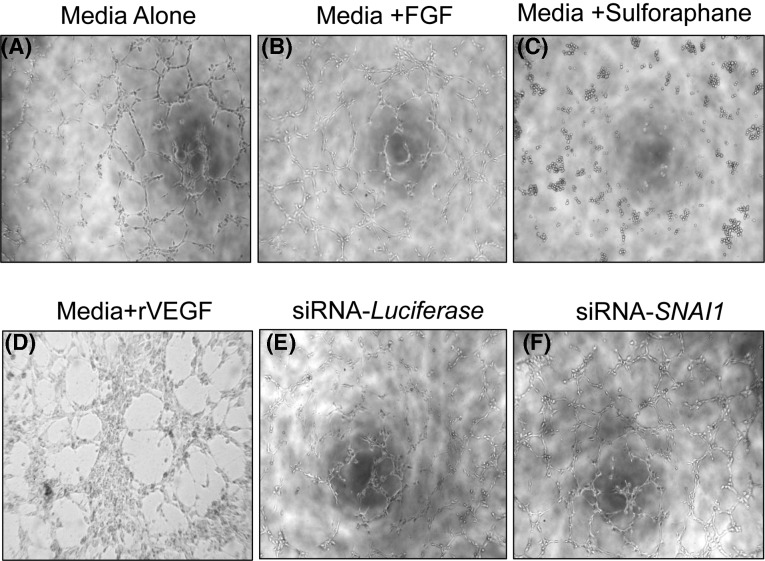
*SNAI1* expression is not required for tube formation in MSCs *in vitro* (**A–D**) MSCs were under different treatments and tested for neoangiogenesis by tube formation assay (*n*=5). (**E,F**) MSCs were transiently transfected with SNA1 siRNA (siSNA1) at a concentration of 50 nM and scrambled siRNA (scr) was used as a negative control. Tube formation was performed with different treatments after *SNAI1* silencing in TDECs and MSCs (*n*=5).

**Figure 4:**
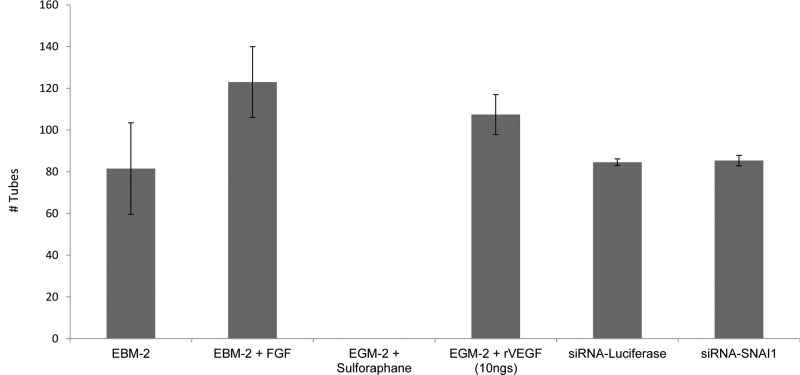
Quantitation of *in vitro* tube formation in MSCs confirmed that *SNAI1* is required for tube formation only in TDECs Quantitation of tube formation in indicated conditions in MSCs was shown. Data are present with means ± S.D. *P<0.05.

To determine if the observed effects of *SNAI1* on tube formation were a global phenomenon, the same assay was conducted in normal mesenchymal stem cells (MSCs). *SNAI1* silencing did not have any effect on tube formation in MSCs indicating that the effect of *SNAI1* is limited to TDECs (Figure 3A–F). The difference between tube formation in MSCs silenced for *SNAI1* or control was shown in Figure 4.

**Figure 5:**
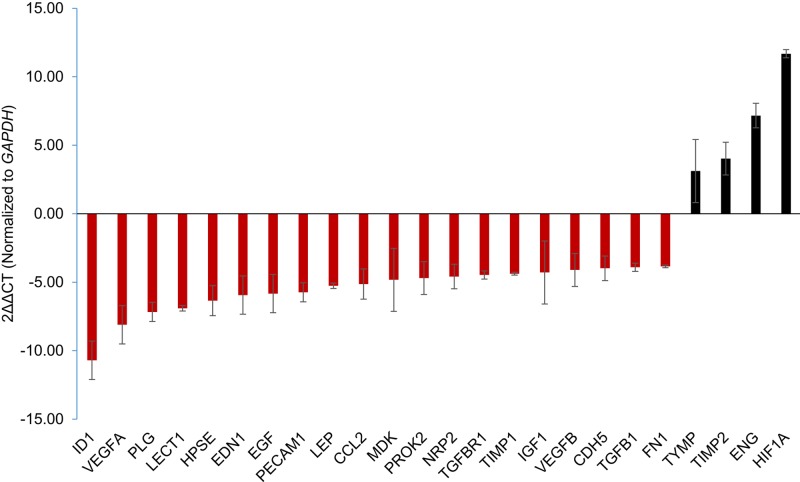
*SNAI1* silencing in TDECs modulates expression of various participants of neoangiogenesis qRT-PCR was utilized to evaluate the expression of angiogenic factors in TDECs transfected with *SNA1* siRNA (*n*=3). Data are present with means ± S.D.

The expression of angiogenic factors was further detected to analyze their connections with Snai1. It was found that a total of 24 genes (21 down-regulated and three up-regulated) had a change more than 3-fold (Figure 5) after silencing *SNAI1. TIMP2, ENG, and HIF1A* were up-regulated 3-fold or higher in TDECs. Amongst the 21 down-regulated genes, *ID1, VEGFA, PLG, LECT1, HPSE*, were the most repressed. Thus, we speculate that *ID1, VEGFA, PLG, LECT1, HPSE* may facilitate with *SNAI1* for neoangiogenesis, and *TIMP2, ENG, and HIF1A* may have the opposite effects.

## Discussion

EMT plays an important role in both developmental and pathological conditions. Variants of traditional EMT include endothelial-to-mesenchymal transition (EndoMT) and partial EMT/EndoMT. Governed by a similar set of signaling and transcription factors, EndoMT contributes to heart valve formation and the generation of cancer-associated fibroblasts, more importantly angiogenesis [11–13]. Various signal molecules, such as HIF-1a, TGF-β2, Notch and Netrin-1, have been reported to be involved in EndoMT [14–16]. Recently P38-GSK3β-Snail signaling pathway induces EndoMT via HMGB1 [17]; however, the role of Snai1 in lung cancer angiogensis is largely unknown. In the present study, we found overexpressed Snai1 in TDECs contributed to tube formation *in vitro*, which may mimic neoangiogenic potential *in vivo*. Previous study has shown that EMT triggers the expression of soluble mediators in cancer cells that stimulate angiogenesis and recruit myeloid cells, which might in turn favor cancer spread [18]. However, the related soluble mediators are mainly IL-8, IL-6, sICAM-1, PAI-1, and GM-CSF. Our study for the first time indicated that snail is a regulator of this process in lung cancer, which provides prospective evidence for the importance of Snai1 in neoangiogenesis.

One possible role of Snai1 in neoangiogenesis may rely on the vascular pattern which is independent of EMT in hepatocellular carcinoma [19]. It indicated that Snai1-mediated EMT could contribute to neoangiogenesis in case where metastasis is dependent on vascular pattern rather than EMT. It has been confirmed that EMT was responsible for chemoresistance but not metastasis in lung and pancreatic cancer [20,21]. It will be important to determine if in these cases tumor metastasis relies largely on vascular patterning as observed in hepatocellular carcinoma.

In conclusion, our study indicated the important role of snail in neoangiogenesis. Furthermore, the effects of other prominent EMT inducers should also be evaluated in the process of neoangiogenesis to further understand the associations between neoangiogenic factors and EndoMT, which may have an exert on EndoMT as well as tumor metastasis.

## Abbreviations

EMT: epithelial to mesenchymal transition
EndoMT: endothelial-to-mesenchymal transition
FGF: fibroblast growth factor
HIF1α: hypoxia-inducible factor 1α
MSC: mesenchymal stem cell
siRNA: small interfering RNA
TDEC: tumor derived endothelial cell
